# Molecular Targeted Approaches for Advanced *BRAF* V600, *N-RAS*, *c-KIT*, and *GNAQ* Melanomas

**DOI:** 10.1155/2014/671283

**Published:** 2014-01-23

**Authors:** Ponti Giovanni, Pellacani Giovanni, Tomasi Aldo, Loschi Pietro, Luppi Gabriele, Gelsomino Fabio, Longo Caterina

**Affiliations:** ^1^Department of Clinical and Diagnostic Medicine and Public Health, University of Modena and Reggio Emilia, Via del Pozzo 71, 41100 Modena, Italy; ^2^Department of Dermatology, University of Modena and Reggio Emilia, Via del Pozzo 71, 41100 Modena, Italy; ^3^Department of Plastic and Reconstructive Surgery, University of Modena and Reggio Emilia, Via del Pozzo 71, 41100 Modena, Italy; ^4^Department of Medical Oncology, University of Modena and Reggio Emilia, Via del Pozzo 71, 41100 Modena, Italy; ^5^Dermatology & Skin Cancer Unit, Arcispedale Santa Maria Nuova, IRCCS, Reggio Emilia, Italy

## Abstract

The introduction of a newly developed target therapy for metastatic melanomas poses the challenge to have a good molecular stratification of those patients who may benefit from this therapeutic option. Practically, BRAF mutation status (V600E) is commonly screened although other non-V600E mutations (i.e., K-R-M-D) could be found in some patients who respond to therapy equally to the patients harboring V600E mutations. Furthermore, other mutations, namely, N-RAS, KIT, and GNAQ, should be sequenced according to distinct melanoma specific subtypes and clinical aspects. In our report, a practical flow chart is described along with our experience in this field.

After decades of unsatisfactory treatments for advanced melanoma, in the last five years, new treatment modalities have been explored that dramatically change the current clinic scenario. The introduction of targeted therapies for melanoma is based on the discovery of genes that are linked to the initiation, progression, and invasion of the tumor [1]. More specifically, somatic mutations in the *BRAF*, *NRAS*, *KIT*, and *GNAQ* genes are critical to correctly stage and manage patients with metastatic disease who can nowadays benefit from these modern molecular targeted therapies. The mutations affect receptor tyrosine kinases and the MAPK and MTOR pathways display different frequencies in distinct histopathological subtypes of melanoma [2].

Somatic mutations in BRAF have been found in almost 50% of all melanomas [3, 4] and most commonly in melanomas derived from skin without chronic sun-induced damage [5]. The result of these mutations (mainly V600E) is enhanced BRAF kinase activity and increased phosphorylation of downstream targets, particularly MEK.

In particular, BRAF inhibitors, targeting the common V600E mutations, have become increasingly popular since they have a high objective response rate and few side effects.

In a previous study we demonstrated that patients harboring uncommon BRAF V600R-M-D mutations, not included in the original experimental protocols of BRAF selective inhibitors, were the responders to the therapy. Surprisingly, patients harboring non-V600E BRAF mutations revealed an objective clinical response similar to V600E melanoma patients [6, 7].

In the clinical setting,* BRAF* mutations are routinely screened but when *BRAF* mutation is not detected, melanomas should be screened for *N-RAS*, *KIT*, and *GNAQ* mutations.

RAS genes are mutated in up to 20% of melanomas which are typically thicker and have a higher mitotic rate [8]. Higher frequency of *KIT* mutation in melanoma is associated with older patients and the acral and mucosal melanoma subtypes [8]. Somatic mutations in the *GNAQ* and *GNA11* genes are found in 80% of uveal melanomas [9]. Nowadays, patients with* N-RAS*, *KIT*, and *GNAQ* mutated tumors can be enrolled in clinical trials of specific inhibitors [2, 8–11].

In the experience of our institution, thirty-two *BRAF* mutated melanomas (32%) were detected among 99 melanomas screened for genetic mutations. Among *BRAF* mutation-negative melanomas, 6 *N-RAS* mutations (four Q61R, one Q61K, and one Q61L) and 3 *KIT* mutations (N822K) were found. The lower *BRAF* mutation rate found in our study compared to the literature might be due to a selection bias since we screened only patients with metastatic disease.

Hot spot V600E mutations were found in 27 patients. V600R mutation and double (V600E-V600M) mutation were identified in two melanomas. In five cases, V600K mutations were found. Two screening failures were noted.

Twenty-three patients with *BRAF* mutated metastatic melanoma were enrolled in the protocol with BRAF inhibitors for compassionate use at the University of Modena. Two *N-RAS* mutated patients were enrolled in an alternative anti-*NRAS* protocol in another University. Mean progression-free survival for *BRAF* positive patients at followup of 8 weeks was 7.6 months (Table 1) (Figure 1). There was no statistically significant difference in the duration of the objective tumor response among different *BRAF* status groupings. An objective response with few side effects was observed in all except one patient (Table 2).

Based on our preliminary findings, we propose a stepwise model to characterize the mutational status of melanomas.

1. Screen for V600E BRAF mutation in melanoma patients with advanced disease (i.e., unresectable stages III and IV) as well as those at high risk of disease progression (stages IIIB and IIIC).
2. In case of negative-V600E *BRAF* mutation, look for other non-V600E *BRAF* mutations (i.e., K, M, R, D).
3. Melanomas not showing* BRAF* mutations should be investigated for *N-RAS* mutations.
4. Double-negative *BRAF* and *N-RAS* melanomas should be further explored for *KIT* mutations or amplifications. This is even more relevant for acral and mucosal melanomas that should be investigated for both *BRAF* and *KIT* mutations at the first step.
5. Triple-negative melanomas may benefit from *GNAQ* mutation evaluation, especially for uveal melanoma.

For melanoma, like other cancers, tailored therapies are dramatically changing the current approaches for treating patients with metastatic disease. However, the heterogeneous molecular defects in melanoma account for the development of drug resistance and thus the different clinical objective responses of targeted therapies. It is known that resistance to *BRAF* inhibitors is due to either the acquisition of secondary mutations in the *BRAF* gene or upregulation of other molecular pathways such as platelet-derived growth factor receptor *β* or *N-RAS*, the consequences of which lead to resistance to MEK and ERK inhibitors [12, 13]. Independent research teams have identified three mechanisms by which melanoma can develop resistance to *BRAF* inhibitors [13, 14]. The findings suggest that *BRAF* inhibitors will need to be combined with other types of drugs, although future studies will have to determine the relative frequency of each mechanism. To conclude, future efforts will be directed not only to develop multitargeted therapies (i.e., *BRAF* and *MEK* inhibitors) but also to further investigate the combination of target treatments and promising immune-therapy approach.

## Figures and Tables

**Figure 1:**
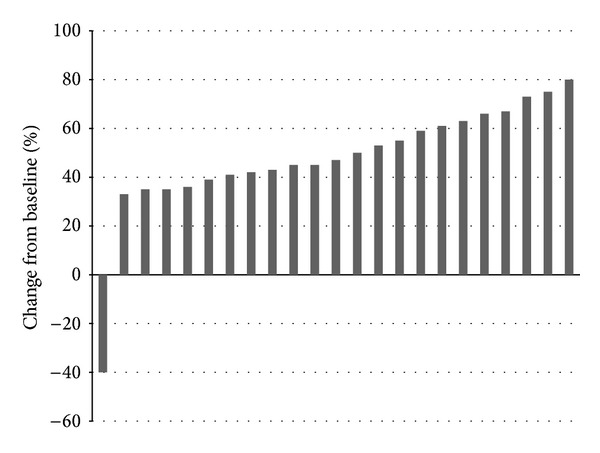
Clinical features of patients treated with *BRAF* inhibitors.

**Table 1:** Patients treated with *BRAF* and *NRAS* inhibitors.

Patient ID	Gender	Age	Treatment duration (months)	Objective response	Time to progression (months)	Followup (months)	Status
1	F	60	6	Partial	6	18	Dead
2	F	61	26	In response	26	29	Alive
3	F	66	4	Partial	4	13	Dead
4	F	51	14	Partial	14	19	Alive
5	M	59	4	Partial	4	5	Dead
6	F	67	6	Partial	6	7	Dead
7	M	51	6	Partial	6	18	Alive
8	M	70	6	Partial	6	11	Dead
9	F	68	23	Partial	In response	23	Alive
10	F	62	6	Partial	6	11	Alive
11	F	81	8	Partial	7	8	Dead
12	M	56	2	None	3	3	Dead
13	F	51	5	Partial	5	5	Dead
14	F	58	18	Partial	In response	18	Alive
15	M	68	6	Partial	14	6	Dead
16	M	43	9	Partial	3	16	Dead
17	M	62	6	Partial	In response	6	Alive
18	M	58	8	Partial	In response	18	Dead
19	M	38	2	Partial	2	3	Dead
20	M	66	12	Partial	10	12	Alive
21	M	65	6	Partial	In response	6	Alive
22	M	79	7	In response	Stable disease	7	Alive
23	M	75	8	Partial	6	8	Dead
1 N-ras	M	69	10	Partial	10	12	Alive
2 N-ras	M	56	6	Stable	6	7	Alive

**Table 2:** Patients treated with *BRAF* inhibitors: frequencies of side effects.

Side effects	Frequency (%)
Arthralgia	54%
Nausea	34%
Skin erythema	28%
Vomiting	14%
Headache	13%
Fatigue	11%
Keratoacanthomas	2%
Hypertransaminasemia	2%
Alopecia	1%
QTc prolongation	1%
